# Incidental Diagnosis of Pseudomyxoma Peritonei: A Case Report

**DOI:** 10.7759/cureus.23425

**Published:** 2022-03-23

**Authors:** Giuseppe Sarpietro, Marco Iraci Sareri, Giulia Maria Bonanno, Maria Grazia Matarazzo, Antonio Cianci

**Affiliations:** 1 General Surgery and Medical Surgical Specialties, Gynecological Clinic, University of Catania, Catania, ITA

**Keywords:** laparoscopy, ascites, appendiceal tumor, pseudomyxoma peritonei, case report

## Abstract

Pseudomyxoma peritonei (PMP) is a rare clinical condition characterized by disseminating gelatinous ascites within the peritoneal cavity with mucinous implants on peritoneal surfaces. We present the case of a patient incidentally diagnosed after laparoscopy: definitive diagnosis after the histological examination was PMP. A 37-year-old female patient with a medical history of infertility and mild pelvic pain was found to have several collections in the pelvis and an amount of free fluid into the Douglas pouch at ultrasound examination. The patient underwent laparoscopic surgical exploration. Peritoneal biopsies and appendectomy were performed. Histological examination was about a low-grade appendiceal mucinous tumor limited to the mucosa without submucosal infiltration with perforation of the wall and deposit of periappendicular acellular mucin. The patient was discharged in good health and referred to an oncological peritoneal center where cytoreductive surgery (CRS) and hyperthermic intraperitoneal chemotherapy (HIPEC) were performed. In conclusion, PMP is an uncommon disease within the abdomen, characterized by a mucinous tumor that produces progressive mucinous ascites. It is characterized by various non-specific symptoms and signs and difficult imaging diagnoses. Histological diagnosis is a determinant to establish the therapy that can differ significantly, depending on the stage of the disease.

## Introduction

Pseudomyxoma peritonei (PMP) is a clinical condition characterized by the dissemination of intra-abdominal gelatinous ascites with mucinous implants on peritoneal surfaces [1]. It is an uncommon disease with an estimated incidence of one to two cases per million per year with a preponderance of female sex. Smeenk et al. reported an annual incidence of PMP approaching two cases per million; they concluded that the incidence of PMP seems to be higher than thought before [2]. 

Generally, PMP arises from an appendiceal mucinous neoplasm; tumor cells produce copious extracellular mucin that can infiltrate the peritoneum. The incidence of appendiceal neoplasm in appendectomies is estimated at 1% [3]. Primary mucinous tumors can arise from other different sites such as the ovary and pancreas; rarely, other sites are primarily involved (gallbladder, stomach, colorectum, fallopian tube, urachus, lung, and breast) [4].

Pseudomyxoma peritonei is characterized by a variety of non-specific symptoms and signs such as abdominal distension, abdominal pain, ascites, and intestinal obstruction. A large number of diagnoses are established coincidentally during a surgical procedure. Clinical symptoms are associated with poorer survival. Other prognostic factors include serum tumor markers and histological findings. Usually, the natural progression of this clinical condition is moderately slow although the course of the disease is related to the histological type of neoplasia [5].

The current gold standard is complete cytoreductive surgery (CRS) followed by HIPEC [6].

## Case presentation

The patient is a 37-year-old female with a medical history of infertility for three years and mild pelvic pain. There was no relevant surgical, family, drug, or allergic history. The patient had no history of malignancy. A pelvic ultrasound examination revealed several collections in the pelvis and an amount of free fluid into the Douglas pouch. The left ovary was surrounded by a collection of suspected pelvic inflammatory diseases. Inflammatory markers, including serum C-reactive protein (CRP) levels, were normal; cancer antigen-125 (CA-125) serum level was 77 UI/mL, carcinoembryonic antigen (CEA) and CA 19-9 levels were normal. Due to the clinical features, laboratory findings, and ultrasound examination, the patient underwent laparoscopic surgical exploration.

**Figure 1:**
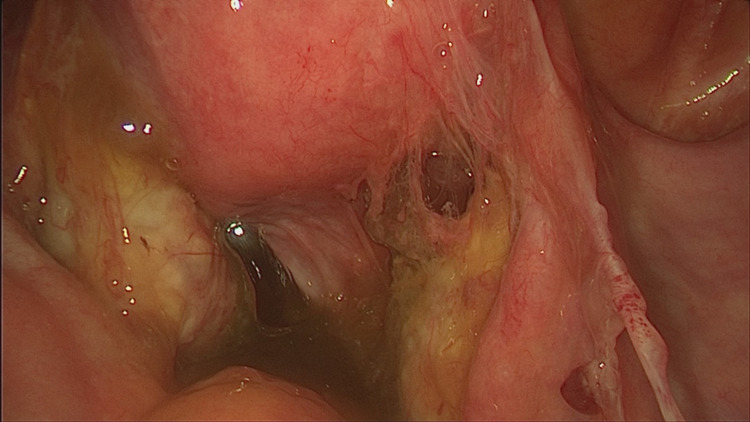
Screen capture of abundant mucinous material in the peritoneal cavity.

Laparoscopy showed unexpected mucus deposits on the peritoneal surfaces; this abundant mucinous material covered the uterus, both ovaries, pouch of Douglas, right and left paracolic gutters, diaphragm dome, and liver surface (Figure 1).

**Figure 2:**
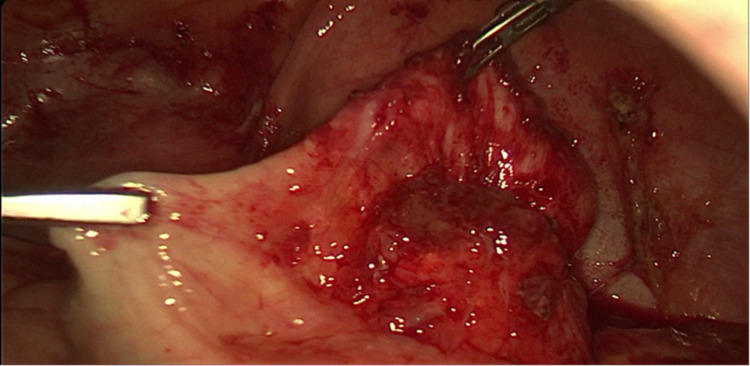
Screen capture of the appendix dilated with perforated walls from which came out gelatinous material.

Uterus and both ovaries had normal morphology and size. The appendix was retro-cecal and dilated with perforated walls from which came out gelatinous material (Figure 2). 

**Figure 3:**
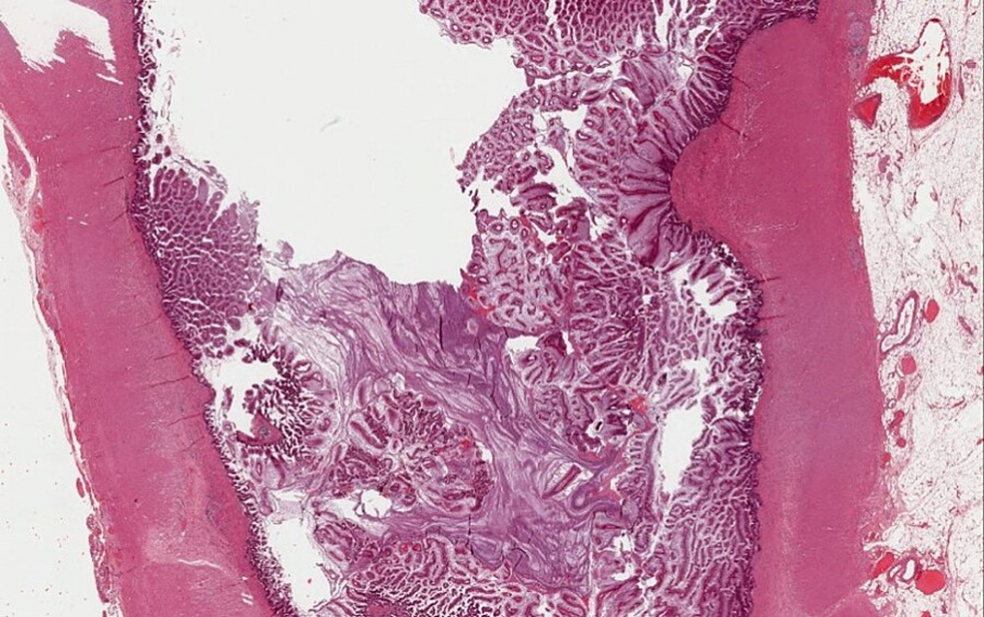
LAMN in cross section: villous or occasionally flat proliferation of mucinous epithelial cells originating from appendiceal lumen. LAMN, low grade appendiceal mucinous neoplasm

**Figure 4:**
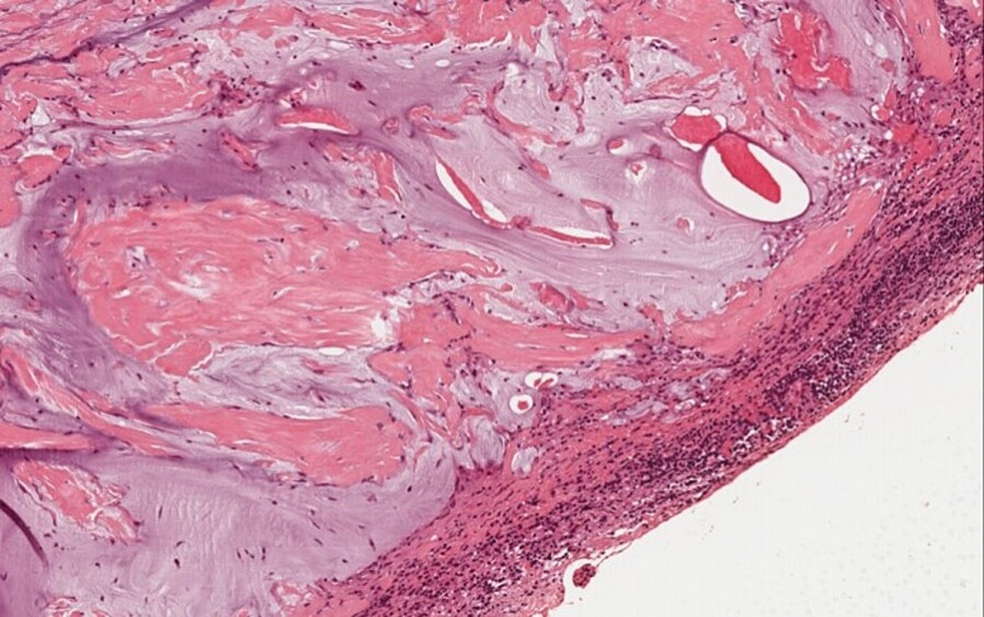
Extra-appendiceal acellular mucin with serosal inflammatory reaction.

No other abnormalities were found in the abdominopelvic cavity. During the procedure, peritoneal lavage was carried out. Peritoneal biopsies and appendectomy were performed. The appendix and peritoneal biopsies were sent to the anatomical pathology department for evaluation. Pathology reported a low-grade appendiceal mucinous tumor limited to the mucosa without submucosal infiltration (low grade appendiceal mucinous neoplasm, LAMN), without vascular invasion, with perforation of the wall and deposits of periappendicular acellular mucin (Figure 3). Peritoneal biopsies showed deposits of mucus material with macrophages, reactive mesothelial cells in a mucus substratum without visible tumor epithelial cells. Peritoneal washing was without atypical cells (Figure 4).

After three days of hospitalization, the patient was discharged in good health conditions with no exceptional medication requirements or complications. Then, the patient was referred to an oncological peritoneal center where CRS and HIPEC were performed.

## Discussion

Pseudomyxoma peritonei is an uncommon disease characterized by the accumulation of mucinous ascites within the peritoneal cavity. In 1842 it was described by Rokitansky as a reaction of the peritoneum secondary to an ovarian neoplasm. Subsequently, in 1884, Werth coined the term PMP and reported the syndrome to be related to an ovarian tumor [7]. PMP term should only be used for the macroscopic aspect of mucinous ascites, as it is not a histological diagnosis.

Generally, mucinous appendiceal neoplasms are the leading cause of PMP; other sites are the ovaries, colon, urachus, and pancreas [8]. Adenomucinous neoplastic cells produce a large amount of mucus and, with progressive growth, eventually cause obstruction of the appendiceal lumen. The perforation of the appendix may lead to the proliferation and production of mucinous ascites. The characteristic pattern of distribution of PMP in the abdominopelvic cavity is due to gravity and movements of peritoneal fluid in the abdomen leading to accumulation of mucous in some regions such as retrohepatic, and rectovesical pouch and omentum giving the characteristic “scalloping effect” although not specific for PMP.

The diagnosis of PMP is challenging. The clinical signs and symptoms are varied and are commonly misdiagnosed as other conditions in the preoperative evaluation. As in our case, about 50% of all cases are asymptomatic and detected incidentally. Other cases can present with abdominal pain, weight loss, nausea and vomiting, a palpable mass, acute appendicitis, intussusception, and localized rupture of abdominal spread. In women, infertility may be a presenting symptom because of ovarian and pelvic involvement by PMP. Surgical treatment with CRS, including bilateral salpingo-oophorectomy, and HIPEC cause iatrogenic menopause.

The ultrasonographic diagnosis is very difficult, it must be suspected, when ascitic fluid is highly echogenic, with immobile echogenic septations with a marked laminated appearance (the ‘onion-skin’ effect) that reflects the concentric layering of mucin, typical of viscous or gelatinous fluid [8]. The gold standard for imaging is CT, preferably with a contrast medium [1].

A variety of non-specific symptoms and signs was described in patients with PMP. Esquivel and Sugarbaker reported in their study that suspected appendicitis was the most common presentation and it accounted for 27% of the cases while abdominal pain was the most common symptom. Furthermore, the authors noted that about 44% of the women had a diagnosis of PMP confirmed while being evaluated for an ovarian mass [9]. Histological diagnosis is determinant to establish the therapy that can differ significantly, depending on the stage of the disease. Our patient was diagnosed with a low-grade appendiceal neoplasm suggesting a satisfactory long-term survival outcome with a low risk of major complications associated with HIPEC. The standard treatment of PMP is a CRS to obtain macroscopic tumor excision combined with HIPEC to treat the microscopic residual disease [6]. Multiple extra-abdominal or retroperitoneal lymph node metastasis during preoperative assessment represents contraindications for HIPEC and CRS. 

In contrast to HIPEC, there is an innovative intraperitoneal chemotherapy approach for peritoneal carcinomatosis, named pressurized intraperitoneal aerosol chemotherapy (PIPAC), developed as a therapy aimed for patients with unresectable peritoneal metastases: intraperitoneal drugs are delivered by utilizing aerosolization and the use of high intraabdominal pressure, produced during laparoscopy, enhancing tissue penetration.

After cytoreduction and HIPEC, the prognosis of PMP is primarily dependent on pathological and biological features. Understanding the underlying molecular mechanisms may facilitate personalized therapy for patients with PMP. KRAS and GNAS mutations are mutations frequently involved in the development of PMP; according to some authors KRAS mutation is detected in 58%-94% of cases [10-11]. Noguchi et al. reported that GNAS mutation is more commonly described in low grade PMP while KRAS mutation is among both low and high-grade PMP; moreover, the activation of the GNAS gene may induce mucin production seen in PMP [11]. Instead, TP53 or PI3K‐AKT mutations may have a crucial role in the progression of PMP [12]. A mutation in TP53 gene is associated to a worse prognosis. There is insufficient evidence about systemic chemotherapy for PMP, however, systemic therapy is a clinical need for unresectable or relapsed peritoneal pseudomyxoma. Pietrantonio et al. evaluated that FOLFOX-4, 5-fluorouracil, and oxaliplatin chemotherapy are tolerable and active in patients with unresectable peritoneal or relapsed pseudomyxoma after standard treatment [13]. According to Hiraide et al., FOLFOX6 which is recently being used more commonly than FOLFOX4, might be an effective and tolerable treatment option for patients with unresectable PMP; they concluded that a combination regimen of 5-FU and oxaliplatin including mFOLFOX6 may be an effective treatment option for patients with unresectable PMP [14]. The optimal therapy for PMP combines complete CRS with HIPEC; it can offer favorable overall survival.

A systematic review and meta-analysis in 2013 concluded that CRS and HIPEC improve the survival of patients with PMP although this treatment strategy is associated with an increased risk of morbidity [15]. Five-year survival after CRS and HIPEC is 60%-100% for low-grade disease and 0%-60% for high-grade disease. Five-year median survival is 79.5%, and 10-year median survival is 55.9% as reported in a systemic review conducted in 2013. For PMP from the appendiceal origin, histological grade could be the only independent prognostic factor.

## Conclusions

Pseudomyxoma peritonei is an uncommon disease. Patients may be asymptomatic or they can present nonspecific and confusing symptoms. The CRS combined with HIPEC as a standard treatment for PMP represents the gold standard therapy. Histological diagnosis is determinant to establish the therapy. Incidental lesions during surgical procedures need to run multiple biopsies, appendectomy, and after pathological diagnosis proceed with adequate treatment in a referral center.
